# Community-based SARS-CoV-2 testing in low-income neighbourhoods in Rotterdam: Results from a pilot study

**DOI:** 10.7189/jogh.12.05042

**Published:** 2022-10-01

**Authors:** Martijn Vink, Zsófia Iglói, Ewout B Fanoy, Janko van Beek, Timo Boelsums, Miranda de Graaf, Helene A.C.M. Voeten, Richard Molenkamp, Marion PG Koopmans, Fraukje EF Mevissen

**Affiliations:** 1 Public Health Service (GGD) Rotterdam-Rijnmond, Rotterdam, the Netherlands; 2 Department of Viroscience, Erasmus MC, Rotterdam, the Netherlands

## Abstract

**Background:**

High incidence of severe acute respiratory syndrome coronavirus 2 (SARS-CoV-2) and low testing uptake were reported in low-income neighbourhoods in Rotterdam. We aimed to improve willingness and access to testing by introducing community-based test facilities, and to evaluate the effectiveness of a rapid antigen detection test (RDT).

**Methods:**

Two to eleven test facilities operated consecutively in three low-income neighbourhoods in Rotterdam, offering the options of walk-in or appointments. Background characteristics were collected at intake and one nasopharyngeal swab was taken and processed using both RDT and reverse transcription polymerase chain reaction (RT-PCR). Visitors were asked to join a survey for evaluation purposes.

**Results:**

In total, 19 773 visitors were tested – 9662 (48.9%) without an appointment. Walk-in visitors were older, lived more often in the proximity of the test facilities, and reported coronavirus disease (COVID-19)-related symptoms less often than by-appointment visitors. For 67.7% of the visitors, this was the first time they got tested. A total of 1211 (6.1%) tested SARS-CoV-2-positive with RT-PCR, of whom 309 (25.5%) were asymptomatic. Test uptake increased among residents of the pilot neighbourhoods, especially in the older age groups, compared to people living in comparable neighbourhoods without community-based testing facilities. RDT detected asymptomatic individuals with 71.8% sensitivity, which was acceptable in this high prevalence setting. Visitors reported positive attitudes towards the test facilities and welcomed the easy access.

**Conclusions:**

Offering community-based SARS-CoV-2 testing seems a promising approach for increasing testing uptake among specific populations in low-income neighbourhoods.

Severe acute respiratory syndrome coronavirus 2 (SARS-CoV-2) emerged in 2019 [1] and caused the largest pandemic of modern times [2]. Two years since the beginning of this pandemic, a vast amount of knowledge about the virus has been acquired and numerous diagnostic tests have been developed [3]. Diagnostic capacities and large test facilities have been established in the Netherlands [4]. However, the availability of testing does not necessarily translate into good accessibility and high testing uptake.

In the beginning of November 2020, cases in the Netherlands surged with weekly incidence figures of 232 new cases per 100 000 population [5]. One of the “hot spots” was Rotterdam [5], likely because it is one of the largest cities in the country (650 000 inhabitants) with a diverse ethnic and socioeconomic populations often living in densely populated neighbourhoods and households [6,7]. Within Rotterdam, the highest proportions of SARS-CoV-2 positive reverse transcription polymerase chain reaction (RT-PCR)-tested populations (30%-36%) were seen in low socioeconomic status (SES) neighbourhoods, while the testing uptake in these areas remained relatively low [8]. Moreover, SARS-CoV-2 sewage data from these neighbourhoods suggested that the actual coronavirus disease 2019 (COVID-19) incidence was likely higher than calculated from the notified cases [9,10]. Community key persons reported that specific groups in these neighbourhoods had less access to available testing facilities due to logistical, financial, or language barriers [11].

The relationships between SES status and SARS-CoV-2 testing rates and test positivity rates have been shown in earlier publications [12-16]. Two studies in New York City and one in Chicago found that people from areas with lower SES had themselves tested for SARS-CoV-2 significantly less often than people from wealthier areas, while the test positivity rate in these areas was higher [12,14]. A study in Santiago de Chile found positive associations between low SES neighbourhoods, SARS-CoV-2 test positivity rates and testing delays due to less access to testing facilities [17].

Generally speaking, inhabitants of low SES neighbourhoods in Rotterdam have less access to SARS-CoV-2 testing facilities, as they are located at the outskirts of the city. Long travel distance to testing facilities and a lack of proper transportation can have a negative impact on test willingness [18]. A study by Hernandez et al. [19] showed that implementing local walk-in testing sites in low-income neighbourhoods was a useful intervention to increase testing uptake. Moreover, previous testing experience, especially when the experience is positive, is also related to higher testing intentions and testing uptake [20]. Finally, offering shorter waiting times for results by using rapid antigen detection tests (RDTs), both for symptomatic and asymptomatic people, may further increase testing rates [21].

To address the accessibility barriers, we piloted community-based testing facilities in three of the most affected low SES neighbourhoods in Rotterdam. We aimed to improve accessibility to testing and therewith to increase testing uptake, not only by establishing community-based facilities, but also by providing walk-in testing options alongside standard scheduled appointment options. To further increase accessibility, individuals with and without COVID-related symptoms could get tested. We hypothesized that increasing accessibility to testing by offering community-based, walk-in testing facilities for everybody regardless of COVID-19-related symptoms, would increase testing uptake in the three selected neighbourhoods in Rotterdam as compared to similar neighbourhoods not offering such testing facilities. Evaluating the efficiency of testing asymptomatic residents was also included. Next to increasing testing uptake, we wanted to explore whether the walk-in option, as compared to testing by appointment, addressed a different public. People may have different preferences regarding health screening such as testing [16]. Knowing these differences can help with tailoring health facilities to individual needs, which in turn can lead to an increase in testing uptake. We hypothesized that the walk-in option may especially serve people with a low SES or an older age [16].

Additionally, visitors’ experiences with the community-based testing facilities and with SARS-CoV-2 testing were evaluated. We hypothesized that people would appreciate the community-based testing facilities. A positive evaluation of our facilities can increase future testing intentions, which in turn provides a good argument to further implement community-based testing facilities.

Finally, the effectiveness of RDTs for detecting asymptomatic SARS-CoV-2 infections was assessed, as it was unknown at the time of the study. Having effective RDTs available can further increase accessibility to testing and testing uptake.

## METHODS

### Design

A cross-sectional quantitative research design was used.

### Setting

At the time of our pilot study (November 24, 2020 to March 5, 2021) standard testing procedures involved making an appointment by phone or online, selecting the date, time, and location. This required either having COVID-19-related symptoms or having had contact with a COVID-19 patient or returning from a high-risk country. Testing was offered for free by municipal health centres at specific SARS-CoV-2 testing locations located at the city outskirts. Visitors testing positive were contacted by the Public Health Service Contact Tracing team (PHS CT-team) through personal phone calls, to perform source and contact tracing and to advise about isolation, quarantine of contacts, and testing measures.

**Figure 1:**
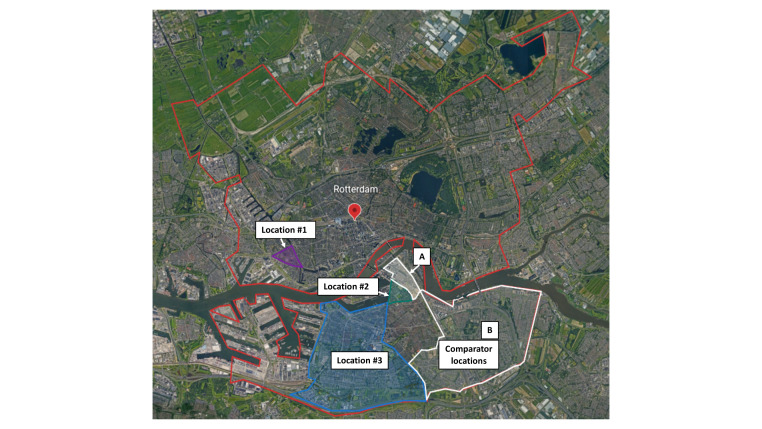
Map of Rotterdam, with the pilot locations 1, 2 and 3 and the comparator locations A and B highlighted (derived from Google maps).

The pilot ran in three different neighbourhoods consecutively in the city of Rotterdam **(****Figure 1****)**. Two testing facilities were established for two weeks at a central square in neighbourhood 1 (location 1: Tussendijken, a district in northeast Rotterdam, population size = 7330; 74% living on a low household income, 65% non-western migrant background [6,22]). Then, two testing facilities were set up at a central square in neighbourhood 2 (location 2: Afrikaanderwijk, a district in southern Rotterdam, population size = 8778; 76% living on a low household income, 80% non-western migrant background) for two weeks. From January until March 2021, 11 testing facilities were placed throughout neighbourhood 3 (location 3: Charlois, a borough in southern Rotterdam, population size = 68 560; 63% living on a low household income, 48% non-western migrant background).

The community-based testing facilities were located at easily accessible and visible spots (eg, close to shopping areas, markets, underground stations, and other public transportation stops). Residents were informed about the testing facilities through social and local media, door-to-door flyers, and posters in multiple languages like Dutch, English, Arab, and Turkish. Also, information was disseminated by general practitioners, key figures, and community groups. The information stated the locations and opening hours of the testing facilities. All residents, both with and without COVID-19-related symptoms, were invited for a free SARS-CoV-2 test. Visitors could choose either to walk in spontaneously (location 1-3) or to schedule their appointment following the standard procedures as described above (location 2 and 3 only). All test results were communicated by e-mail and/or by phone (see procedures as described above).

### Procedure

Upon entry at the testing facility, routine data were collected from each visitor. After that, one nasopharyngeal swab was taken. Upon exit at location 1 and 2, visitors were asked by a PHS employee to join a ten-minute survey to evaluate their experience with the testing facility.

### Data sources, variables, and measurements

In the Netherlands, SARS-CoV-2 is a notifiable infection. This indicates that routine data are collected nationally and stored in protected databases. A selective download from this database was shared with the lead author, anonymized, and used for evaluating testing uptake and background characteristics of people getting tested in our pilot neighbourhoods. The data source used for analyses included age, gender, presence of COVID-19- related symptoms, postal code, test date, test location, and test result.

The survey included questions on background characteristics (age, gender, self-reported ethnicity, household size, and presence of COVID-19-related symptoms) and experiences with the SARS-CoV-2 testing procedure and testing facilities (number of previous tests, test reason, attitude towards the test, intention for future testing, knowledge about/attitude towards the testing facility, and location preference for future testing). Survey data were also used for evaluating the background characteristics of people getting tested and their testing experiences.

From each visitor, one nasopharyngeal swab, provided by the SD Biosensor SARS-CoV-2 Rapid Antigen Test (distributed by Roche, Basel, Switzerland) (REF No. 9901-NCOV-01G; LOT No QCO3020096/Sub:A-4), was taken. This swab was placed in an empty tube and transported to the mobile on-site laboratory. Swabs were immediately processed for the RDT, following manufacturer’s instructions. To enable RT-PCR testing on the same material, a non-standard protocol was followed; leftover RDT material (approximately 200μl from the original 350μl) was added to 1ml non-supplemented Dulbecco's Modified Eagle Medium (DMEM), to ensure sample stability (unpublished results), and shipped to the Erasmus MC Viroscience laboratory for RT-PCR, using 500μl volume in the Cobas® SARS-CoV-2 test on the COBAS 6800 system (Roche diagnostics, Basel, Switzerland). Test data were used for evaluating the effectiveness of the RDT for detecting asymptomatic SARS-CoV-2 infections.

### Analyses and statistical methods

To learn if the pilot had an impact on test uptake, we compared the total number of people, residing in one of the three locations, that had themselves tested (in any testing facility/location) to the total number of residents of two control areas in Rotterdam that had themselves tested. These control areas had comparable population sizes and household incomes. Pilot locations 1 and 2 were compared with comparator location A (Feijenoord district: population = 7610; 77% living on a low household income), pilot location 3 was compared with comparator location B (IJsselmonde borough: population = 61 360; 55% living on a low household income) (**Figure 1**).

Appointment- and location-specific data for all visitors were analysed for associations with sex, residence, presence of COVID-19-related symptoms (using χ^2^ tests), and age (using Wilcoxon rank-sum tests). To evaluate the efficiency of our approach (inviting non-symptomatic people), test positivity rates for those with and without symptoms were compared. These data were analysed with STATA (version 15.1; StataCorp, College Station TX, USA).

Background characteristics of survey participants at location 1 (walk-in only) and 2 (walk-in and by-appointment) were compared to evaluate if the walk-in option served an additional segment of the population. Additionally, experiences with the testing procedure and testing facility were compared to evaluate if walk-in only visitors (location 1) evaluated the testing facilities differently than a combination of walk-in and by-appointment visitors. Frequency analyses were used for reporting total numbers; *t* tests and χ^2^ tests were performed to compare the responses of participants at the two locations, using SPSS (version 26; IBM, Armonk NY, USA). See Appendix 1 in the **Online Supplementary Document** for the survey guide.

To evaluate the performance of the RDT, especially amongst asymptomatic positive visitors, SARS-CoV-2 RDT results were compared to RT-PCR results (in location 1 and 2). Confidence intervals were calculated with the Wilson method. SARS-CoV-2 diagnosis in locations 1 and 2 was based on the RT-PCR results, while in location 3 it was based on the RDT results, following the positive outcomes of the evaluation in locations 1 and 2.

### Ethics

Ethical approval was not requested since this study was performed as a public health intervention in an outbreak situation and as part of a response to a public health emergency. Testing was voluntary and was carried out for diagnostic purposes, and the use of leftover material for assay validation is permitted under Dutch law. All data collection procedures in our study were performed in accordance with the principles of the Declaration of Helsinki.

The routine data collected among visitors of the testing facilities, as part of national COVID-19 surveillance policy, were stored in password protected national databases (see https://ggdghor.nl/privacystatement-coronavirus-testing/). A selective download from this database was shared with the lead author and anonymized before use in further analyses. For the survey data, informed consent was obtained from all interviewed individuals at the testing sites and these data were collected anonymously (no personal information like name, address, or phone number was collected). In addition, participation in the survey was voluntary and participants were not required to answer questions they did not feel comfortable with.

## RESULTS

### Testing uptake

During the pilot period, 4.6% of the total population of the three boroughs had themselves tested in one of the facilities. This percentage varied between 1.3% at location 2, 1.7% at location 1 and 11.4% in location 3 **(****Table 1****)**.

**Table 1:** Background characteristics of pop-up testing facility visitors, differentiated by location and appointment type

	Location 1			Location 2				Location 3				Total							
	**Walk-in**			**Walk-in**		**Appointment**		**Walk-in**		**Appointment**		**Walk-in**			**Appointment**				
	**n**		**%**	**n**	**%**	**n**	**%**	**n**	**%**	**n**	**%**	**n**	**%**	**n**			**%**	Statistical test result*	
**Number of visitors**	1750	100		1159	53.2	1021	46.8	6753	42.6	9090	57.4	9,662	48.9	10 111			51.1		
**Gender**																			
Male	932	53.3		589	50.8	479	46,9	3329	48,2	4468	49.2	4841	50.1	4947			48.9	3.8	
Female	814	46.5		565	48.8	541	53.0	3359	49.7	4577	50.4	4738	49.0	5118			50.6		
Unknown	4	0.2		5	0.4	1	0.1	74	1.1	45	0.5	83	0.9	46			0.5		
**Median age (interquartile range)**	50.9 (33.7-62.9)			49.3 (32.9-62.2)		30.5 (24.5-43.1		50.8 (31.6-65.9)		34.1 (25.8-49.0)		50.7 (32.3-65.0)		33.7 (25.6-48.5)				40.7†	
**Visitors with COVID-19-related** **symptoms**	805	46.0		588	50.7	875	85.7	1666	24.7	6775	74.5	3,059	31.7	7650			75.7	3853†	
**Visitors from the neighbourhood**	1307	74.7		719	62.0	254	24.9	5188	76.8	2654	29.2	7,214	74.7	2908			28.8	4167†	
**Percentage of the neighbourhood’s population that was tested‡**	1.7			1.3				11.4				4.6							

*For all comparisons, χ^2^ tests were used, except for the median age, where the Wilcoxon rank-sum test was used.

†*P*<0.001.

‡Combined figure for walk-in and by-appointment visitors.

**Figure 2:**
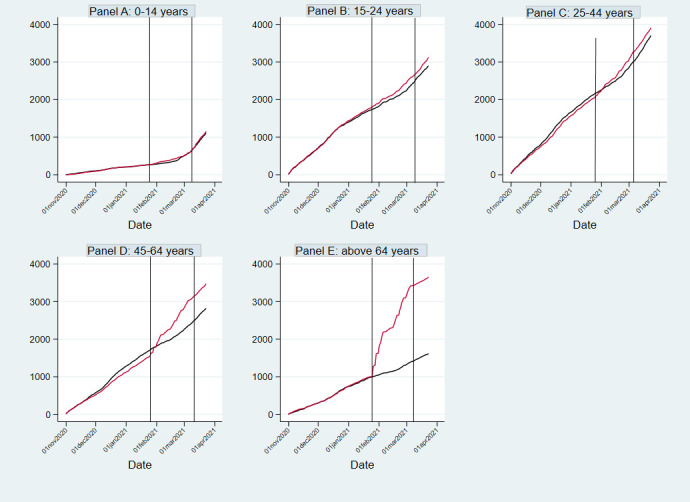
Cumulative number of residents of location 3 (Charlois) and comparator location B (IJsselmonde) having undergone a SARS-CoV-2 test, per 10,000 population. The area between the vertical lines indicates the 6-week intervention period in location 3 (Charlois). For reasons of simplicity, this figure only shows the number of SARS-CoV-2 tests from November 1, 2020 onwards. The red line indicates location 3 (Charlois), while the blue line indicates the comparator location B (IJsselmonde).

**Figure 2** shows the cumulative number of SARS-CoV-2 tests in location 3 and in the comparator location B, subdivided by age group. In the two oldest age groups (45-64 years (**Figure 2**, panel D) and above 64 years (**Figure 2**, panel E)) in location 3, this figure rose sharply during the pilot period, while the increase remained constant in the comparator location. In locations 1 and 2 we saw comparable trends with sharply increasing numbers of SARS-CoV-2 tests (especially in the oldest age groups), while the increase remained constant in the comparator location A (Figures S1 and S2 in the **Online Supplementary Document**). In the period after the pilot the figures in all three locations again showed similar trends as in their comparator locations.

### Background characteristics of walk-in vs on appointment visitors

Of the 19 773 people tested, 9662 (48.9%) visited the testing facilities without an appointment (walk-in), while 10 111 (51.1%) came on appointment. No significant gender differences between these two groups of visitors were found. The median age of the walk-in visitors (50.7 years) was significantly higher than of the visitors who came by appointment (33.7 years; *P* < 0.001). 31.7% of the walk-in visitors reported COVID-19-related symptoms, as opposed to 75.7% of the by-appointment visitors (*P* < 0.001). Walk-in visitors came significantly more often from the borough where the testing facilities were located (74.7%) than visitors who came on appointment (28.8%; *P* < 0.001). These differences between the two groups of visitors were visible both in location 2 and 3 **(****Table 1****)**.

### Test results and COVID-19-related symptoms

In total, 1211 visitors (6.1%) tested positive. Test positivity was higher among the visitors who came by appointment (7.5%) than among the walk-in visitors (4.6%, *P* < 0.001). Significant differences were found in test positivity between visitors with and without COVID-19-related symptoms (8.4% and 3.4% respectively, *P* < 0.001); these differences were also visible in the subgroups defined by appointment type, although among the walk-in visitors this difference was larger (8.7% vs 2.7%, compared to 8.3% vs 5.2% for the by-appointment visitors) **(****Table 2****)**.

**Table 2:** SARS-CoV-2 positivity rate of pop-up testing facility visitors, differentiated by location and appointment type

	Location 1		Location 2					Location 3						Total							
	**Walk-in**		**Walk-in**			**Appointment**		**Walk-in**		**Appointment**				**Walk-in**		**Appointment**		**All visitors**		**Statistical test result**	
	**n**	**%**	**n**	**%**		**n**	**%**	**n**	**%**	**n**			**%**	**n**	**%**	**n**	**%**	**n**	**%**		
**SARS-CoV-2 positives**	134	7.6	96	8.3		71	6.9	218	3.2	692			7.6	448	4.6	763	7.5	1,211	6.1	73.1*†	
**Test positivity per sex**																					
Male	64	6.8	50		8.5	36	7.5	107	3.2	343		7.7		221	4.6	379	7.7	600	6.1	0.0088‡	
Female	69	8.4	46		8.1	35	6.5	108	3.2	343		7.5		223	4.7	378	7.4	601	6.1		
Unknown	1	25.0	0		0	0	0	4	5.4	6		13.3		5	6.0	6	13.0	11	8.5		
**Test positivity per age group**																					
0-14 years	8	11.8	5		11.4	2	22.2	10	3.5	38	8.7			23	5.8	40	9.0	63	7.4	16.2*§	
15-24 years	19	12.0	11		9.7	11	5.0	31	5.2	108	8.0			61	7.0	119	7.5	180	7.3	28.1*§	
25-44 years	49	10.4	32		9.9	36	6.6	64	3.3	284	6.4			145	5.3	320	6.5	465	6.0	16.5*§	
45-64 years	38	5.8	29		7.0	16	8.4	64	3.2	209	10.1			131	4.3	225	10.0	356	6.7	24.9*§	
65 years and above	20	5.0	18		7.1	6	11.3	47	2.5	49	6.4			85	3.4	55	6.8	140	4.1		
Unknown	0	0	1		12.5	0	0	2	20.0	4	9.1			3	16.7	4	8.7	7	10.9	N/A	
**Test positivity by presence of COVID-19-related symptoms**																					
Yes	92	11.4	66		11.2	65	7.4	109	6.5	570	8.4			267	8.7	635	8.3	902	8.4	219.9*‖	
No	42	4.5	30		5.3	6	4.1	109	2.1	122	5.3			181	2.7	128	5.2	309	3.4		
Unknown	0	0	0		0	0	0	0	0	0	0			0	0	0	0	0	0		

*P<0.001.

†χ^2^ test comparing test positivity between the two appointment groups.

‡χ^2^ test comparing test positivity between the total groups of males and females (excluding visitors for which sex was unknown).

§χ^2^ test comparing test positivity between all visitors in specific age group with the reference age group (65 years and above).

‖χ^2^ test comparing test positivity between the total groups of visitors with and without COVID-related symptoms (excluding visitors for which the symptoms were unknown).

### Background characteristics of survey participants (walk-in vs by appointment)

**Table 3** provides an overview of the background characteristics of the survey participants differentiated by location 1 (walk-in only) and location 2 (walk-in and by-appointment). Compared to location 1, participants at location 2 were significantly younger (*P*  = 0.020), more often female (*P*  = 0.041), and more often self-reporting a Dutch ethnicity, as opposed to a non-Dutch ethnicity (ie, Turkish, Surinam, Moroccan, Cape Verdean, or other) (*P*  = 0.003). Participants at location 2 more often reported COVID-related symptoms (*P*  = 0.030). No significant differences in household size were found.

**Table 3:** Background characteristics of survey participants differentiated by location

Characteristic	n*		Mean (SD)		Median		Range		%		Statistical test results†
**Location**	**1**	**2**	**1**	**2**	**1**	**2**	**1**	**2**	**1**	**2**	
**Age**											
Reported	384	191	49.2 (18.0)	44.3 (18.2)	51.5	44.0	11-87	10-99			3.1‡
Missing	8	4									
**Gender**											
Male	197	81							50.3	42.4	4.2§
Female	186	110							47.4	57.6	
Missing	9	4									
**Self-reported ethnicity**											
Dutch	143	96							36.8	49.5	8.7§
Non-Dutch	246	98							63.2	50.5	
Missing	3	1									
**Household Size**											
Reported	376	192	2.32	2.42	2.0	2.0	0-8	0-12			-0.7
Missing	16	3									
**COVID-19-related symptoms**											
Yes	166	101							42.3	52.1	4.7‡
No	224	93							57.1	47.9	4.7‡
Missing	2	1									

*The number of participants is not equal for each characteristic as it was not obligatory for them to answer all questions.

†For all comparisons, χ^2^ tests were used, except for the mean age and the mean household size, where t-tests were used.

‡*P*<0.05.

§*P*<0.01.

### Evaluating SARS-CoV-2 testing experiences and community-based testing facilities among visitors

In general, participants were very positive about the testing facilities. A vast majority stated it was a (very) good idea (99.7%), (very) important (97.2%), and (very) nice (92.3%). Participants at location 1 (walk-in only) were significantly more positive about the testing facilities than those at location 2 (walk-in & by-appointment; *P*  = 0.002). Furthermore, participants at location 1 significantly more often reported knowing about the community-based testing facilities from neighbours or by word of mouth (*P*  = 0.004) or by coincidently passing by, as compared to participants at location 2 (*P* = 0.01), while those at location 2 more often reported other sources of information (*P* < 0.0001). Most participants indicated that it was the first time they got tested (66.8%, n = 385); this percentage was slightly higher at location 1 than at location 2 (*P*  = 0.045). Participants gave different reasons for getting tested, with “to be sure”, “having COVID-19 related symptoms”, and “having had contact with a confirmed positive person” mentioned as the most important motivations. The majority intended to be tested again (81.5%, n = 466). The community-based testing facilities were preferred by participants at location 1 while participants at location 2 more often had no preference (*P* < 0.0001). Participants at location 1 more often supported their location preference because of short travel distance (*P*  = 0.016) and not needing an appointment (*P*  = 0.011) as compared to participants at location 2 (**Table 4**).

**Table 4:** Evaluation of pop-up testing facilities and SARS-CoV-2 testing, differentiated by location

Variable	n		Mean (SD)		%		Statistical test result*
**Location**	**1**	**2**	**1**	**2**	**1**	**2**	
**Attitude towards facility†**							
Reported	379	190	4.4 (0.46)	4.3 (0.44)			3.2‡
Missing	13	5					
**How did you know about the facility?**							
Neighbours, word of mouth	114	34			29.2	18.1	8.3‡
Coincidently passing	104	32			26.7	17.0	6.6‡
Social media	75	36			19.2	19.1	0.0
Family/housemates	66	22			16.9	11.7	2.7
Community organizations	15	11			3.8	5.9	1.2
Other§	52	71			13.3	37.8	45.2‖
Missing	2	7					
**First time testing**							
Yes	268	117			68.4	60.0	4.0¶
No	117	74			29.8	37.9	
Missing	7	4					
**Reason for testing**							
To be sure	197	69			51.8	35.6	13.7‖
COVID-19-related symptoms	124	95			32.6	49.0	14.5‖
Contact	44	33			11.6	17.0	3.3
Housemate with COVID-19	8	9			2.1	4.6	2.9
Other**	50	21			13.2	10.8	0.7
Missing	197	69			51.8	35.6	
**Future test intentions**							
Yes	322	144			83.6	77.0	5.8¶
Maybe	50	29			13.0	15.5	
No	13	14			3.4	7.5	
Missing	6						
**Location preference**							
Local testing facility	332	138			88.5	77.1	17.3‖
Standard test street	4	11			1.1	6.1	
No preference	39	30			10.4	16.8	
Missing	17	16					
**Location argument**							
Travel distance	261	108			74.1	63.9	5.8
No appointment needed	175	64			49.7	37.9	6.5‡
Not meeting acquaintances	10	3			2.8	1.8	0.5
Other††	46	27			13.1	16.0	0.8
Missing	40	26					

*For all comparisons, χ^2^ tests were used, except for the attitude towards the facility (through Likert scale), where t-tests were used.

†Likert scale 1-5; 3 items; α = 0.67 (n = 587).

‡*P*<0.01.

§E.g., GP, school, flyer.

‖*P*<0.001.

¶*P*<0.05.

**Reasons mentioned were: for holidays, GP, school, taking responsibility, high infection rates in neighbourhood, contact with vulnerable people, personal vulnerability, work-related high contact, curiosity.

††Arguments mentioned were: having no personal transport, scheduling did not work, faster, low-key, drive-in is more comfortable with bad weather.

### Performance of the rapid antigen test amongst people with or without symptoms

Compared to RT-PCR, overall sensitivity of the RDT was 84.1%, while specificity was 99.0%. Sensitivity was higher amongst people with symptoms than among asymptomatic people (88.3% vs 71.8%). This can be explained by the significant difference of RT-PCR Cycle threshold (Ct) values between the two categories (mean Ct = 30.2 vs 32.0; *P* < 0.05). Sensitivity was higher among people with symptom onset less than seven days ago (91.4%) than among people whose symptoms started more than seven days prior (79.2%) (**Table 5**).

**Table 5:** Clinical sensitivity and specificity of an RDT among pop-up testing facility visitors, differentiated by symptoms

	Symptomatic	Asymptomatic	0-3 days since onset	0-7 days since onset			>7 days since onset	Overall
**Clinical Sensitivity (95% CI)**	88.3% (83.4-92.2)	71.8% (60.5-81.4)	95.0% (90.4-97.8)	91.4% (87.0-94.8)			79.2% (57.9-92.9)	84.1% (79-4-88.0)
n	223	78	160	222			18	301
**Clinical Specificity (95% CI)**	98.9% (98.3-99.3)	99.2% (98.7-99.6)						99.0% (98.7-99.3)
n	2027	1571						3598

## DISCUSSION

Easily accessible SARS-CoV-2 testing facilities are, next to COVID-19 vaccinations, the cornerstone of the fight against the pandemic. By introducing walk-in testing facilities, in addition to the standard by-appointment option, we aimed to increase testing accessibility and, in turn, testing uptake in low-income neighbourhoods in Rotterdam with relatively high SARS-CoV-2 test positivity rates. The pilot was evaluated by analysing testing rates, visitor characteristics, visitors’ experiences, and the usefulness of the RDT for detecting asymptomatic SARS-CoV-2 infections. Of the total population in the three pilot areas, 4.6% got tested in one of the testing facilities, enabling us to achieve a sharp increase in testing uptake, especially in the age groups above 44 years. The testing facilities were evaluated positively and the RDT showed acceptable sensitivity amongst asymptomatic people, given the high prevalence rates.

Walk-in visitors were older than by-appointment visitors and more often lived in the proximity of the pop-up testing facilities. These community-based testing facilities thus seem to fill the gap for the older, less mobile populations. The survey results underline the finding that walk-in visitors differ from by-appointment visitors: although the survey did not collect appointment-type information, its results show that background characteristics of participants at exclusively walk-in locations differed from those at walk-in plus by-appointment locations. Survey participants from the latter were more often higher educated and reported having an ethnic Dutch background more often. Similar background differences between walk-in vs by-appointment visitors were found in New Orleans [16], where inhabitants above 50 years strongly preferred walk-in testing facilities close to their homes above drive-through testing facilities farther away. Walk-in testing facilities seem to attract a different public than by-appointment test facilities, which shows that offering different types of testing facilities will serve a wider population.

Test positivity was 4.6% in the walk-in group and 7.5% in the by-appointment group. This was lower than the average test positivity (11.3%) found in standard test facilities in Rotterdam during the pilot period [17]. As expected, test positivity was higher among visitors with COVID-related symptoms than among visitors without symptoms. However, among the total SARS-CoV-2 positive visitors, a considerable proportion (25.5%) were asymptomatic. In a mass screening campaign in Italy, the proportion of asymptomatic infections was higher, but this may be due to the higher testing uptake [18]. Interestingly, the percentage of SARS-CoV-2 positive diagnoses amongst asymptomatic visitors was higher in the by-appointment group than in the walk-in group. This may be the result of the national testing criteria at the time of the pilot, stipulating that all contacts of COVID-19 positive persons should schedule a SARS-CoV-2 test. A considerable proportion of these contacts would be asymptomatic at the time of testing.

In the months prior to the pilot, the cumulative age-specific testing figures for the pilot areas and control areas incurred equally, but after its start, the cumulative testing figures for the pilot area rose more sharply, especially in the older age groups. As there were no other differences in the available testing facilities in these two areas, this increase is likely the result of the pilot. Crossover was limited, as only 0.6% of the residents of the control borough got themselves tested in the pop-up testing facilities in location 3. It is crucial that we were able to achieve a higher testing uptake among older people, as they have a higher risk to develop serious COVID-19-related complaints [23].

Although it was not an aim of our pilot, we still expected to detect an increased number of SARS-CoV-2 positive residents in our three pilot locations because of our community-based testing facilities. However, in a comparison of SARS-CoV-2 diagnoses between location 3 and comparator location B this was not visible, both during and after the intervention (Figure S3 in the **Online Supplementary Document**). Also, we did not see an impact on the number of infectious people in location 3, as measured in the SARS-CoV2 sewage levels (Figure S4 in the **Online Supplementary Document**). This may be the result of the relatively low testing uptake (4.6%). With a longer pilot period, more testing facilities, and intensified communication strategies, we could have tested more residents and also have seen increased numbers of SARS-CoV-2 positive diagnoses in the pilot areas.

The aim of our pilot was to increase test accessibility among a hard-to-reach population, thereby increasing test uptake. The survey results indicate that we succeeded to create an easy-access testing approach: many people knew about the facilities by spontaneously passing by, and for the majority, it was the first time they got tested, even though they had had opportunities before. Additionally, most participants reported a preference for community-based testing facilities for future testing, because of the short travel distance and the non-necessity to make an appointment. Walk-in sites in New Orleans also increased testing uptake for specific populations (African American, Asian, and elderly) because of their proximity [19]. Moreover, Bevan et al. [24] and McElfish et al. [25] found that low accessibility (due to distance and absence of private transport) was one of the main barriers to getting tested for SARS-CoV-2. The positive experiences reported by our visitors may also help in removing potential test barriers, such as fear of the test procedure [17] and developing a positive attitude towards testing and positive intentions for future testing [26,27]. Low-key easily accessible testing facilities can thus contribute to higher testing uptake, especially among vulnerable people.

At the time of the study, neither the proportion of asymptomatic SARS-CoV-2 positive persons nor the RDT sensitivity in this group was known. The test performed below the 80% sensitivity threshold set by World Health Organization (WHO) [28], but better than in another study investigating close contacts at day five post-contact [29]. By now, it is well established that sensitivity is directly related with viral load, which is highest in the first seven days after symptom onset. This can only be estimated from the date of contact (if it is known), making this a challenge for asymptomatic SARS-CoV-2 positives due to their lack of symptoms. Therefore, even though below the 80% threshold, the RDT can provide useful assistance in finding these asymptomatic cases. Although other diagnostic tests like RT-PCR can potentially be performed in mobile laboratories, logistics and necessary equipment make this more complicated and the time to results still remains longer. The short waiting time for RDT can have a positive effect on people’s test willingness [13]. Moreover, faster results can prevent further transmission and improve willingness to comply with measures until the results are obtained. Given these acceptable outcomes, using RDT among the asymptomatic population was recommended in the national testing policy. Testing uptake was lower than we expected. Turnout in location 3 was higher (11.4%) than in locations 1 (1.7%) and 2 (1.3%), likely due to the longer pilot period and the higher number of test facilities. Although our study shows that testing uptake can be increased by facilitating access to testing, there are many other factors that influence testing motivation and testing uptake [30,31]. Studies in several other countries showed associations between educational level or ethnicity and low testing uptake [16,32]. Others found factors such as perceived discrimination at work, concerns of individual’s privacy, and feelings towards testing to influence test intentions [31]. To be better prepared for future pandemics, it would be important to further explore the reasons for low testing uptake among specific populations.

The study has some limitations. First, our pilot was performed in a real-time setting, during a pandemic situation. Given these circumstances, a randomized controlled trial could not be done. This influences the strength of our conclusions on the effectiveness of our pilot. However, by comparing the data collected among residents in our pilot locations with those of comparable neighbourhoods, we still managed to come to reliable conclusions. Second, survey participants were a selective group of those who got tested during our pilot (22% at location 1 and 9% at location 2); therefore, their responses may not be representative. We aimed for high participation rates and for a representative sample by using a very brief survey, stressing that participation was anonymous, and by including data collectors that could speak various languages representative for the neighbourhoods’ residents. Moreover, survey data were collected at different time points and different days of the week, including the weekend, to further enhance sample diversity and representativeness. Finally, performance testing of RDTs amongst asymptomatic people was based on a relatively small group. However, we could only have included more asymptomatic people by extending our study period, which was not feasible. Although these results were thus more indicative than definitive, they still served as important input for national testing policy, together with a few other reports on RDT performance, which was essential information in high-prevalence, pre-vaccine pandemic times.

## CONCLUSIONS

With pop-up testing facilities, we were able to increase SARS-CoV-2 testing rates in low-income neighbourhoods in Rotterdam and attract additional groups that would probably not have been tested in the standard testing facilities. We believe that community-based testing facilities would also be useful in low-income countries, where access to regular SARS-CoV-2 testing facilities is often limited and where sophisticated laboratories and trained staff required for RT-PCR-based diagnosis are often unavailable [24]. We recommend using a community-based testing approach to reach all layers of the population in both situations.

## Additional material:


Online Supplementary Document
