# Supplementary material for: Community-based SARS-CoV-2 testing in low-income neighbourhoods in Rotterdam: Results from a pilot study
Source: J Glob Health. 2022 Oct 1;12:05042. doi: 10.7189/jogh.12.05042 (PMC9526478; doi:10.7189/jogh.12.05042)
Supplement: Online Supplementary Document [file jogh-12-05042-s001.pdf]

Supplementary figure S1: Cumulative number of residents of Location 1 (Tussendijken) and comparator location A (Feijenoord), having undergone a SARS-CoV-2 test (per 10,000 population)

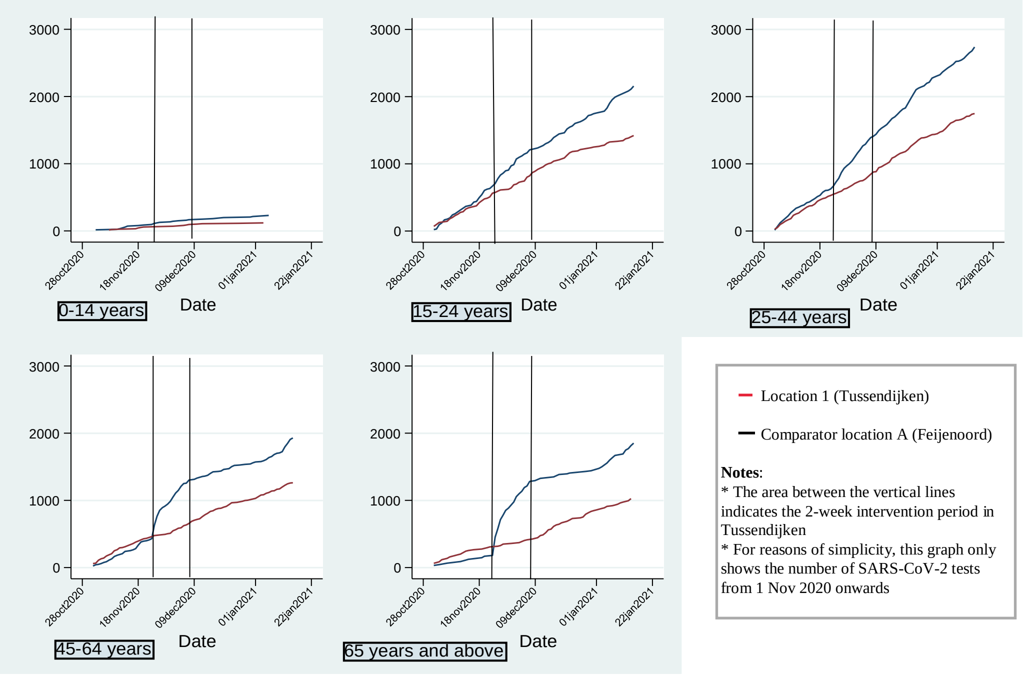

Notes:
* The area between the vertical lines indicates the 2-week intervention period in Tussendijken
* For reasons of simplicity, this graph only shows the number of SARS-CoV-2 tests from 1 Nov 2020 onwards

Supplementary figure S2: Cumulative number of residents of location 2 (Afrikaanderwijk) and comparator location A (Feijenoord), having undergone a SARS-CoV-2 test (per 10,000 population)

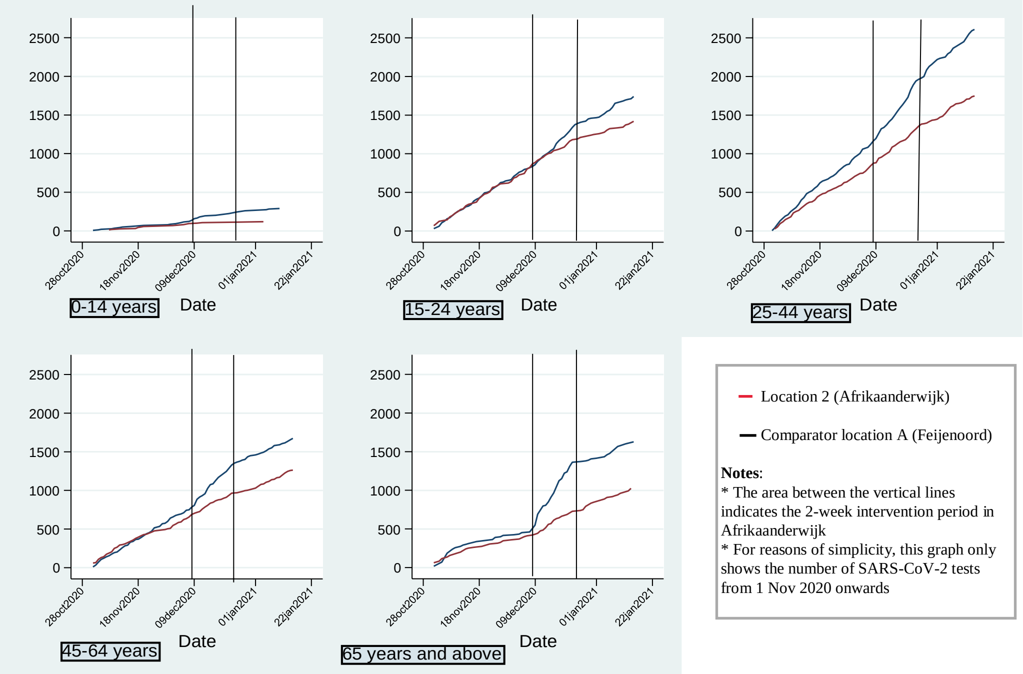

Supplementary figure S3: Cumulative number of residents of location 3 (Charlois) and comparator location B (IJsselmonde), testing SARS-CoV-2 positive (per 10,000 population)

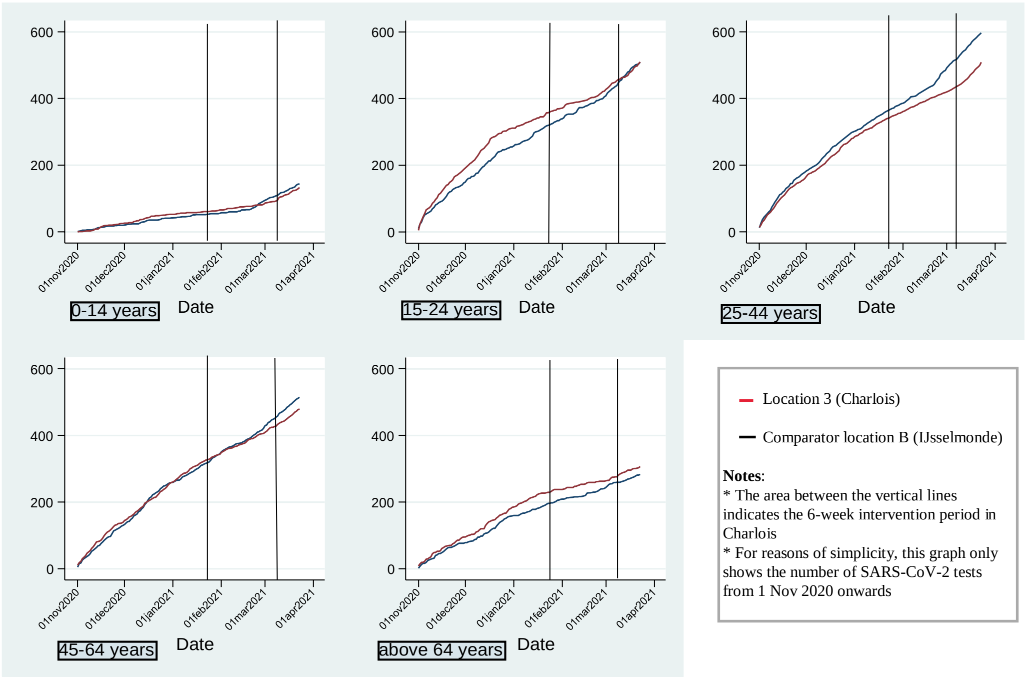

Supplementary Figure S4: SARS-CoV-2 measurements in household sewage water, comparing pilot location 3 (Charlois borough) with two comparison boroughs (Feijenoord and IJsselmonde)

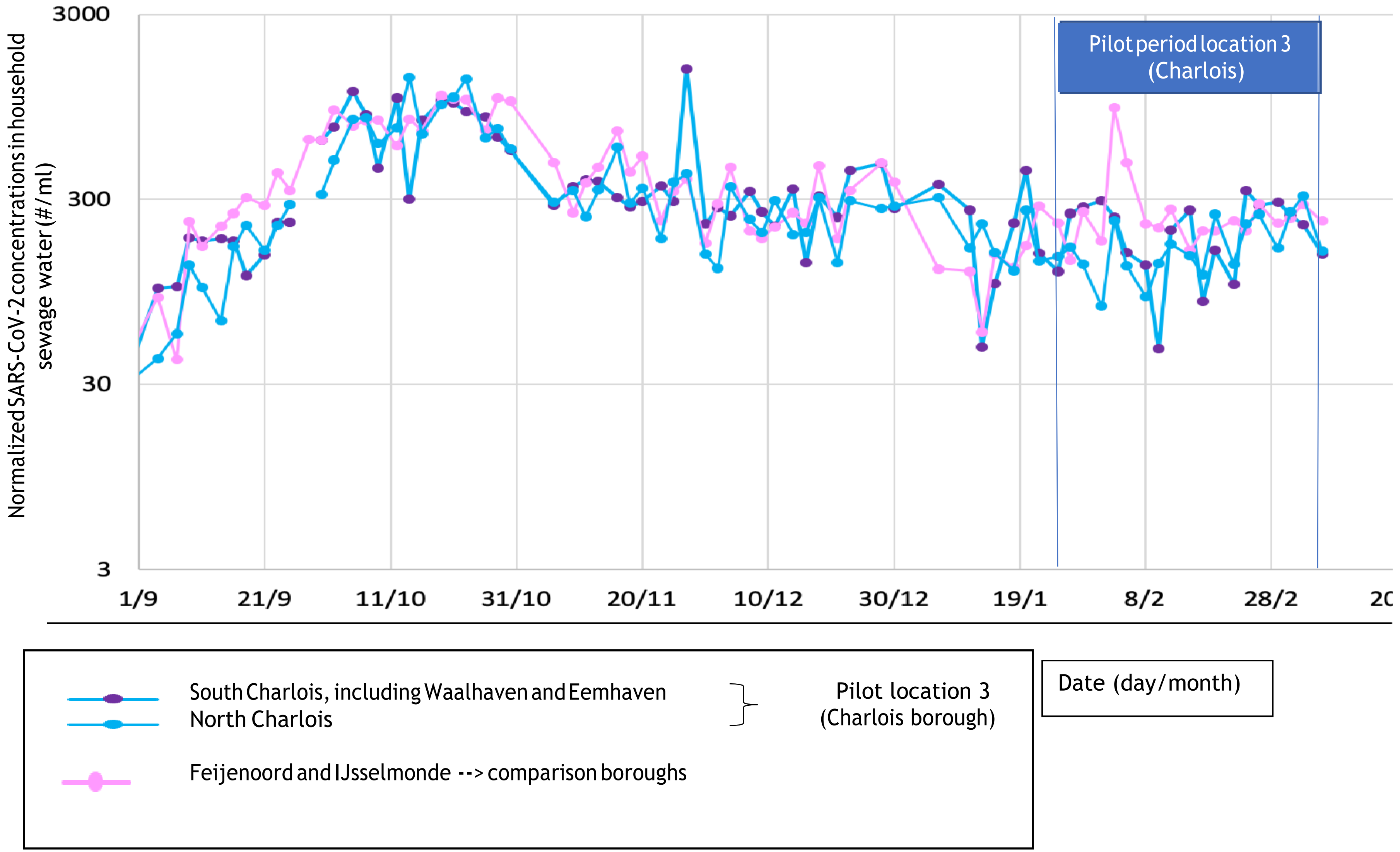

Appendix S1: Survey Evaluation Corona Testing facility Intervention

[English version] The Municipal Health Centre is offering corona-testing in these test-facilities. We would like to hear your opinion regarding these testing-facilities, Corona-testing, and the regulations. Would you like to join?

It takes approximately 10 minutes. Your opinion is important to us. Knowing your opinion will help us to figure out if we are organizing things properly or if things need to be changed. Your participation is anonymous; we won't ask for your names or address or other contact details. You are not obliged to answer questions if you don't want to.

[Dutch version] De GGD is gestart met het aanbieden van corona-testen in deze testbus. Graag zouden wij uw mening horen over deze testbus, over corona-testen en over de maatregelen. Zou u hieraan mee willen doen?

Het duurt ongeveer 10 minuten. Uw mening is belangrijk voor ons. Met uw mening weten we of we goed bezig zijn of dat zaken misschien anders kunnen. Uw deelname is anoniem; wij vragen niet naar uw naam of adres of andere contact gegevens. U bent niet verplicht om vragen te beantwoorden als u dat niet wilt.

Background Variables

Achtergrond Variabelen

1. *May I ask how old you are?*

   Age:

   Mag ik vragen hoe oud u bent?

   Leeftijd:

2. *Select gender* *Male Female*

   Omcirkel geslacht: MAN VROUW

3. *To which ethnicity do you include yourself?*

   Tot welke etniciteit rekent u zichzelf?

   a. *Dutch*
   b. *Moroccan*
   c. *Turkish*
   d. *Cape Verdian*
   e. *Surinam*
   f. *Antillean*
   g. *Other, namely:……*

4. Met hoeveel mensen deelt u een huishouden? Vul in Aantal:

   With how many people are you sharing a household? Enter number:

5. Wat vindt u van dit test-bus initiatief?

   What do you think of this testing-facility initiative?

   De testbus vind ik…

   I *think the testing-facility is …*

   a. *A very bad idea*
   b. *A bad idea*
   c. *Not a bad idea but also not a good idea (neutral)*
   d. *A good idea*
   e. *A very good idea*

6. *I think the testing-facility is …*

a. *Very unimportant*
b. *unimportant*
c. *Not important but also not unimportant (neutral)*
d. *Important*
e. *Very important*

7. *I think the testing-facility is …*
   a. *Very unpleasant*
   b. *unpleasant*
   c. *Not unpleasant but also not pleasant (neutral)*
   d. *Pleasant*
   e. *Very pleasant*

8. Hoe wist u van dit testbus initiatief?

   How did you know about this testing-facility initiative?
   a. Ik liep toevallig langs bus / ik zag de bus hier staan

      I coincidentally passed the facility / i noticed the facility
   b. Gehoord van huisgenoten (familie)

      I heard about it from housemates (family)
   c. Gehoord van buren / buurtgenoten / mond-op-mond

      I heard about it from neighbours / word of mouth
   d. Lokale welzijnsorganisaties, namelijk:

      Local well-being organisations such as…..
   e. Social media, namelijk:

      Social media such as….
   f. Other namely…………

9. Heeft u op dit moment corona-gerelateerde klachten?

   Do you currently have corona related symptoms (complains?)
   a. *Yes*
   b. *No*

10. Is dit de eerste keer dat u zich laten testen?

    Is this the first time you got tested? (yes/no)

11. Waarom heeft u dit keer besloten om u te laten testen? (meerdere antwoorden mogelijk)

Why did you decide this time to get tested? (multiple answers possible)

a. Ik heb corona-gerelateerde klachten

I have corona related symptoms

b. Omdat het via de testbus makkelijker is dan de teststraat

Because it is easier at this test facility than at the testing street

c. Gewoon voor de zekerheid

Just in case

d. Ik heb een huisgenoot met corona

I have a housemate with corona

e. Ik ben in contact geweest met iemand die corona heeft

I have been in contact with somebody with corona

f. Other, namely…….

12. Wat vindt u van de corona-test?

What do you think of the corona test?

I think doing a corona-test is…

a. A very bad idea

b. A bad idea

c. Not a bad idea but also not a good idea (neutral)

d. A good idea

e. A very good idea

13. I think doing a corona test is….

a. Very unimportant

b. unimportant

c. Not important but also not unimportant (neutral)

d. Important

e. Very important

14. I think doing a corona test is…

a. Very unpleasant

b. unpleasant

c. Not unpleasant but also not pleasant (neutral)

d. Pleasant

e. Very pleasant

15. I think doing a corona test is…

a. Very uncomfortable

b. Uncomfortable

c. Not uncomfortable but also not comfortable (neutral)

d. Comfortable

e. Very comfortable

16. Bent u van plan om u nog eens te laten testen (waar dan ook)?
Are you planning to get tested again (where ever)?
    a. *Yes*
    b. *Maybe*
    c. *No*

17. Waar laat u zich liever testen; hier in de testbus of verderop in een test-straat?
Where would you prefer to get tested: Here at this testing facility or further away at a testing street?
    a. Hier in de Test-bus in de buurt
    Here at a local testing facility
    b. Test-straat verderop
    Testing street further away

18. Why? (multiple answers possible)
    a. *Travel distance*
    b. *Not meeting anybody I know*
    c. *You don't need an apopintment* / kunt zo naar binnen *lopen you can enter just like that*
    d. *Other namely: ....*
